# Allergic diseases of the skin and drug allergies – 2019. Higher doses for heavy hives

**DOI:** 10.1186/1939-4551-6-S1-P106

**Published:** 2013-04-23

**Authors:** Kiran Godse

**Affiliations:** 1 Dermatology, DR.D.Y.Patil Medical College and Hospital, India

## Background

Nonsedating antihistamines are recommended as first line treatment for patients with urticaria.

## Methods

In our study, 30 patients with chronic urticaria for at least six weeks were enrolled after an informed written consent. Out of 30 patients 16 were females and 14 were males, in the age group of 16 yrs to 55 yrs (average age 33 yrs) .

Patients with urticaria activity score (UAS) of 3 or more than 3 ,were started on tablet levocetirizine in a dose of 10 mg at bedtime at the onset of the treatment.

Out of 30 patients only 5 patients were symptomatic at the end of one week and were started on 20 mg of levocetirizine. Three out of 5 patients were asymptomatic after starting higher dose of 20 mg at the end of 2^nd^ week with reduction in UAS. Twenty eight out of 30 patients showed good response and decrease in the UAS within two weeks , with higher dose of levocetirizine. However 2 patients showed no response to treatment even with 20 mgs of levocetirizine.

Average UAS at 0 week was 4.767, it came down to 1.8 at the end of one week. At the end of two weeks UAS was 1.4. At the end of 4 weeks average UAS came down to 0.4, showing a marked downward trend with high doses of levocetirizine given at the very onset of the disease. Therefore patients with higher UAS at the time of presentation should be started on a higher dose of levocetirizine at the onset of the treatment for better symptomatic relief and suppression of the disease within one week.

## Results

According to the findings in our study use of a higher dose of non sedating antihistamine in patients with UAS of 3 or more than 3 at the very start of the therapy brings about a better control and rapid supression of the symptoms, suggesting the need for “higher doses for heavy hives.”

## Conclusions

we suggest higher doses for heavy hives

